# Supplementary material for: Binder-Free α-MnO2 Nanowires on Carbon Cloth as Cathode Material for Zinc-Ion Batteries
Source: Int J Mol Sci. 2020 Apr 28;21(9):3113. doi: 10.3390/ijms21093113 (PMC7247688; doi:10.3390/ijms21093113)
Supplement: Supplementary file 1 [file ijms-21-03113-s001.docx]

Article

Binder-Free α-MnO_2_ Nanowires on Carbon Cloth as Cathode Material for Zinc-ion Batteries

Ryan Dula Corpuz ^1^, Lyn Marie De Juan-Corpuz ^1,2,3^, Mai Thanh Nguyen ^4^, Tetsu Yonezawa ^4,5^,
Heng-Liang Wu ^6,7^, Anongnat Somwangthanaroj ^1^, Soorathep Kheawhom ^1,8,^*

^1^ Department of Chemical Engineering, Faculty of Engineering, Chulalongkorn University,
Bangkok 10330, Thailand; ryan.d@chula.ac.th (R.D.C.); lyn.m@chula.ac.th (L.M.D.);
anongnat.s@chula.ac.th (A.S.)

^2^ Research Center for the Natural and Applied Sciences, University of Santo Tomas,
Manila 1015, Philippines

^3^ Department of Chemical Engineering, Faculty of Engineering, University of Santo Tomas,
Manila 1015, Philippines

^4^ Division of Materials Science and Engineering, Faculty of Engineering, Hokkaido University,
Hokkaido 060-8628, Japan; mai_nt@eng.hokudai.ac.jp (M.N.); tetsu@eng.hokudai.ac.jp (T.Y.)

^5^ Institute of Business-Regional Collaborations, Hokkaido University, Hokkaido 001-0021, Japan

^6^ Center for Condensed Matter Sciences, National Taiwan University, Taipei 10617, Taiwan; hengliangwu@ntu.edu.tw

^7^ Center of Atomic Initiative for New Materials, National Taiwan University, Taipei 10617, Taiwan

^8^ Research Unit of Advanced Materials for Energy Storage, Chulalongkorn University,
Bangkok 10330, Thailand

***** Correspondence: soorathep.k@chula.ac.th; Tel.: +66-81-490-5280

Received: 28 February 2020; Accepted: 26 April 2020; Published: 28 April 2020

**Abstract:** Recently, rechargeable zinc-ion batteries (ZIBs) have gained a considerable amount of attention due to their high safety, low toxicity, abundance, and low cost. Traditionally, a composite manganese oxide (MnO_2_) and a conductive carbon having a polymeric binder are used as a positive electrode. In general, a binder is employed to bond all materials together and to prevent detachment and dissolution of the active materials. Herein, the synthesis of α-MnO_2_ nanowires on carbon cloth via a simple one-step hydrothermal process and its electrochemical performance, as a binder-free cathode in aqueous and nonaqueous-based ZIBs, is duly reported. Morphological and elemental analyses reveal a single crystal α-MnO_2_ having homogeneous nanowire morphology with preferential growth along {001}. It is significant that analysis of the electrochemical performance of the α-MnO_2_ nanowires demonstrates more stable capacity and superior cyclability in a dimethyl sulfoxide (DMSO) electrolyte ZIB than in an aqueous electrolyte system. This is because DMSO can prevent irreversible proton insertion as well as unfavorable dendritic zinc deposition. The application of the binder-free α-MnO_2_ nanowires cathode in DMSO can promote follow-up research on the high cyclability of ZIBs.

**Keywords:** zinc; zinc-ion battery; nanowires; single crystal; α-MnO_2_; dimethyl sulfoxide

1. Introduction

Nowadays, due to the increasing use of energy in modern society and intensifying degrees of electrification, rechargeable batteries are in great demand. In particular, lithium-ion batteries (LIBs) are versatile and have a wide range of applications, proving them to be the market leaders. However, LIBs have several shortcomings, such as safety issues, recycling, and especially their high cost and limited resources [1–3]. Therefore, alternative battery technologies using cheap and abundant materials such as sodium (Na) [4,5], aluminum (Al) [6,7], magnesium (Mg) [8–10], and zinc (Zn) [11–13] for electrodes are actively sought after in order to address these concerns.

Zn exhibits favorable low redox potential, high stability, and a high specific volumetric capacity of 5855 mAh cm^-3^, which is even greater than that of lithium (2066 mAh cm^-3^) [14]. These properties make it a promising anode material [15,16]. Zn is low in cost and highly abundant. It has been applied in various types of batteries, such as zinc-nickel [17], zinc-air [18,19], zinc-iodine [20], zinc-iron [21], and zinc-ion [22,23]. In addition, an established Zn recycling industry is available to recover Zn from its myriad of consumptions in different industries [24]. Moreover, since Zn is rather stable in ambient condition, a zinc-ion battery (ZIB) permits easier and inexpensive handling for fabrication and packaging [25].

Different cathode materials have been employed for ZIBs, e.g., Prussian blue analogues [26–28], vanadium-based oxides [29,30], and manganese-based oxides (MnO_x_) [31]. For a long time, MnO_x_ has been a subject of intensive research due to its numerous potential applications in different electrochemical energy storage and conversion devices, such as fuel cells, supercapacitors, and batteries [32–35]. Manganese oxide (MnO_2_) is also considered a potential electrode for ZIBs [36–38]. MnO_2_ is inexpensive and exhibits a high theoretical capacity. Nonetheless, MnO_2_ cathodes are subjected to the challenging issues of poor cyclability [39]. Yet, introduction of MnSO_4_ into the electrolyte was found to be beneficial for enhancing cycling stability since it inhibited MnO_2_ dissolution [40]. Another difficulty confronted in exploiting the MnO_2_ cathode is its poor conductivity which increases the internal resistivity of the ZIBs, resulting in poor performance of the battery [41]. However, a solution frequently applied is the introduction of a conductive agent, such as carbon black, reduced graphene oxide (rGO) [42], and carbon nanotubes (CNTs) [43], which are usually used with polymeric binders to bind all materials together on the current collector. Of late, reducing the content of additives and binders is much in favor in order to accomplish a high mass loading of active material in the battery. It is observed that polymeric binders increase both processing and material costs. Moreover, it is evident that some binders, such as polyvinylidene fluoride (PVDF), require an undesirable toxic solvent such as N-methyl-2-pyrrolidone (NMP) for electrode fabrication [44,45]. Thus, a binder-free MnO_2_/rGO electrode was examined as cathode material for ZIBs [42]. Consequently, the binder-free MnO_2_/rGO demonstrated enhanced capacity, excellent rate capability, and cycling stability in comparison to that of the conventional MnO_2_ electrode. A binder-free δ-MnO_2_-carbon composite electrode was synthesized and applied in ZIBs, and efficient charge transfer and improved cyclability was reported [46].

Research in ZIBs is commonly studied via aqueous electrolytes not only because of their low cost and versatility, but most importantly, because of their high ionic conductivity which favorably enhances battery performance [47]. Nevertheless, when an aqueous electrolyte is used, critical issues such as severe capacity fading resulting from proton insertion [48] and decomposition of water lead to hydrogen gas evolution and unsatisfactory low coulombic efficiency [27]. Therefore, the development of a nonaqueous electrolyte system based on organic solvents and ionic liquids was carried out [49–51]. For instance, over the years, our group has actively studied nonaqueous systems in developing MnO_2_-based ZIBs [46,52]. In fact, strategies to enhance electrochemical performance, cyclability, and stability of ZIBs using a Deep Eutectic Solvent (DES) based on choline chloride/urea/zinc chloride mixture were successfully developed [52]. In previous work, a ZIB using δ-MnO_2_ with nanoflower morphology in DES was demonstrated. In a following study, the capacity, stability, and cyclability of DES-based ZIBs were greatly improved by enhancing the conductivity and surface area of δ-MnO_2_ [46]. This was achieved by developing a binder-free δ-MnO_2_-carbon fiber composite via hydrothermal process coupled with annealing, which resulted in better connectivity of the MnO_2_ network due to the removal of interlayer and physiosorbed water. However, despite this success, long-term stability and cyclability as well as a high capacity could not be attained using the DES electrolyte. Thus, other strategies to improve the nonaqueous ZIBs were implemented.

In this paper, an α-MnO_2_ nanowire-carbon fiber composite was synthesized. Thereby, the electrochemical performance of this novel cathode material was investigated in both aqueous and nonaqueous electrolyte systems. It is a known fact that nanostructured materials show greater surface area and redox reaction sites in comparison to its bulk counterpart. Therefore, it is expected that MnO_2_ with its one-dimensional (1D) nanostructure will show enhanced electrochemical performance.

2. Materials and Methods

2.1. Materials

All chemicals were used as received without further purification: potassium permanganate (KMnO_4_), QRëC, Auckland, New Zealand; ammonium sulfate ((NH_4_)_2_SO_4_), Sigma-Aldrich, St. Louis, MO, USA; carbon cloth (AvCarb 1071 HCB), AvCarb Material Solutions, Lowell, MA, USA; deionized (DI) water; isopropyl alcohol (IPA), Ajax Finechem, Auckland, New Zealand; sulfuric acid (H_2_SO_4_), Ajax Finechem, Auckland, New Zealand; zinc trifluoromethanesulfonate (Zn(OTf)_2_), Sigma-Aldrich, St. Louis, MO, USA; and Dimethyl sulfoxide (DMSO), Sigma-Aldrich, St. Louis, MO, USA.

2.2. Electrode and Battery Fabrication

The carbon cloth was surface treated with 1.0 M H_2_SO_4_ for 1 h, washed with DI water several times, and vacuum dried at 60 °C for 2 h before usage.

In a typical experiment, 0.1264 g KMnO_4_ and 0.0428 g (NH_4_)_2_SO_4_ were dissolved and mixed in 40 mL DI water. Then, the resulting solution was sonicated for 1 h and hydrothermally synthesized at 180 °C for seven days using a Teflon-lined autoclave decorated with carbon cloth on its inner wall. Next, the carbon cloth, deposited with MnO_2_ particles, was washed with DI water several times, rinsed with IPA, and vacuum dried for at least 4 h before usage.

In a typical experiment, pre-cut Zn foil (Shandong AME Energy Co. Ltd., China) 15 mm in diameter and 0.08 mm thick was ultrasonicated in acetone for 30 min. Then, it was washed with distilled water several times and rinsed with IPA before vacuum drying for 2 h. Next, 0.1 M and 3.0 M aqueous Zn(OTf)_2_ and 0.1 M Zn(OTf)_2_ in DMSO were used as electrolytes to investigate the electrochemical performance of the nanowire α-MnO_2_-carbon fiber composite.

A CR2032 cell was used to assemble the ZIB. The assembled battery consisted of an anode (made up of Zn foil), a cathode (hydrothermally grown MnO_2_ on carbon cloth), and an electrolyte (Zn(OTf)_2_ dissolved in DI water or DMSO). The electrodes were separated with a glass microfiber (Whatman, Sigma-Aldrich, St. Louis, MO, USA), punched into a disc which was 19 mm in diameter, and enclosed within circular metal cases. The mass loading of α-MnO_2_ on circular carbon cloth is 1.3 mg/cm^2^.

2.3. Characterization

To evaluate the electrochemical performance of the fabricated Zn/MnO_2_ battery, the following electrochemical tests were conducted: cyclic voltammetry (CV), electrochemical impedance spectroscopy (EIS), and galvanostatic charge-discharge tests.

1. Galvanostatic charge-discharge tests were conducted via Battery Testing System (NEWARE, BTS-4000 series, Neware Technology Ltd., Shenzhen, China) at 30 °C within the voltage range of 0.4–1.9 V. The equivalent C-rate per current density was indicated: 50, 100, 150, and 200 mA g^-1^ correspond to 0.16 C, 0.32 C, 0.49 C, and 0.65 C, respectively.

2. EIS tests were conducted using VersaSTAT 3F (AMETEK, Berwyn, PA, USA) at an amplitude potential of 10 mV around the open circuit voltage (OCV) within the frequency range of 0.01–100,000 Hz.

3. CV tests were conducted using VersaSTAT 3F (AMETEK, Berwyn, PA, USA) within the voltage range of 0.7–2.1 V vs. Zn/Zn^2+^ with scan rates of 0.5, 2.0, 5.0, and 10.0 mV/s. The tests were carried out using the CR2032 cell with a two-electrode configuration. In this configuration, the positive electrode of the cell was used as the working electrode. The negative electrode of the cell was used as both counter and reference electrode.

Both elemental and morphological analyses were investigated using scanning electron microscope (SEM), JEOL (Peabody, MA, USA) JSM-6480LV, 15 kV, with energy dispersive X-Ray spectroscopy (EDS) and transmission electron microscope (TEM), JEOL (Peabody, MA, USA) JEM-1400, 100 kV. The crystalline and phase structure was determined using X-ray diffraction (XRD), Bruker (Billerica, MA, USA) D8-Advance, Cu Kα radiation, λ = 1.5418 Å, operating at 40 kV and 40 mA within 2θ range of 5 to 90 degrees.

3. Results and Discussion

The investigation commenced by optimizing different parameters such as hydrothermal synthesis time and temperature. Figure S1 shows SEM images at different hydrothermal synthesis time, at T = 140 °C. Thus, it is observed that the sample obtained is a mixed morphology of nanoflowers and nanowires, even if synthesis time was prolonged from one to seven days. However, when the hydrothermal processing temperature and time was set to 110 °C and 24 h, respectively, the nanoflower morphology without the presence of nanowires was readily obtained, as shown in Figure S2. On the other hand, when temperature increased from 140 °C to 180 °C while keeping the synthesis time for seven days, the nanowire morphology without the presence of nanoflowers was obtained.

Figure 1 displays the representative image of MnO_2_ nanowires produced at 180 °C for seven days. The nanowires have a diameter 26 ± 5 nm, where the observed thick wires of ~100 nm are bundles of wires. As shown in Figure S2, EDS analysis depicts the elements Mn, O, C, and K, verifying the possible existence of the MnO_2_-carbon fiber composite. To determine the structure and phase of these MnO_2_ nanowires, XRD analysis was conducted.


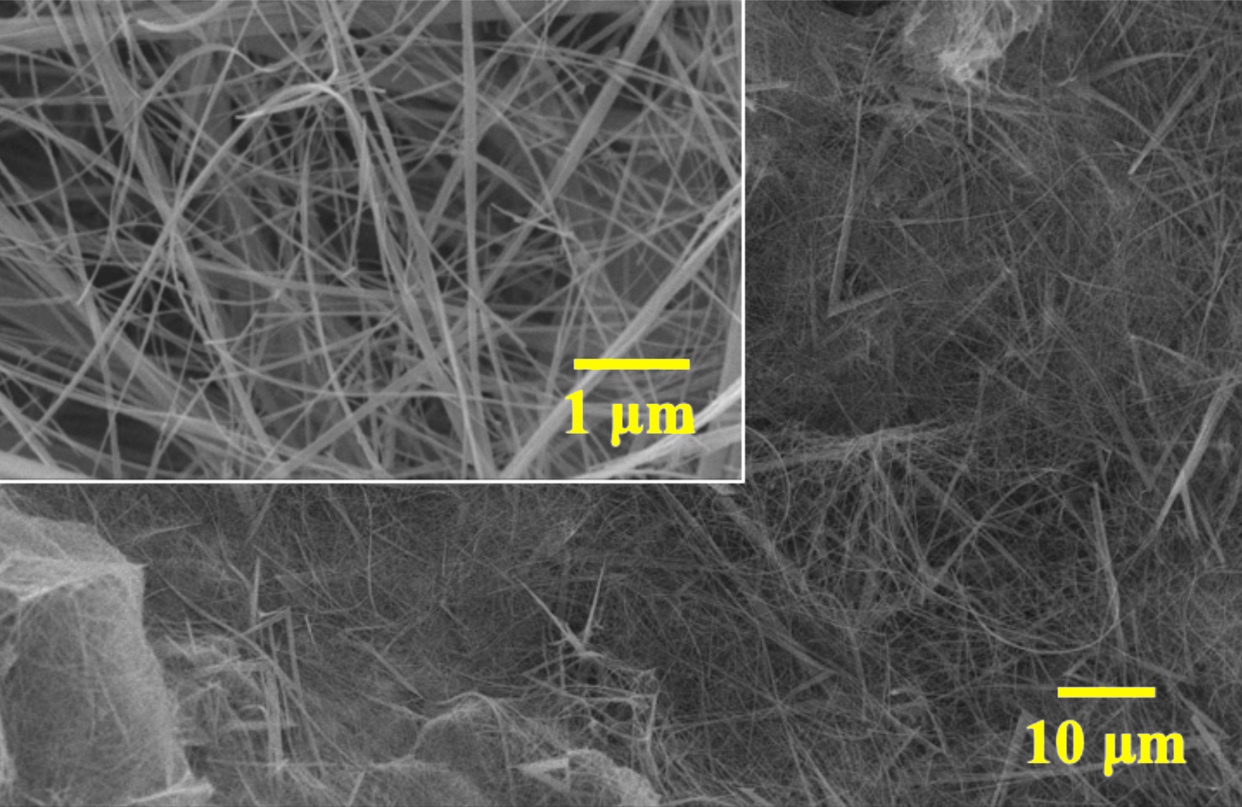


**Figure 1.** SEM image of α-MnO_2_ nanowires hydrothermally synthesized at 180 °C for seven days.

Figure 2a shows the XRD analysis of the MnO_2_-carbon fiber composite. Based on the prominent diffraction peaks at 2$\theta$ $\simeq$ 18.1°, 25.7°, 28.8°, 36.7°, 37.5°, 39.0°, 41.2°, 42.0°, 49.9°, 56.4°, 60.3°, 65.1°, and 69.7°, which correspond to the planes (200), (220), (310), (400), (211), (330), (420), (301), (411), (600), (521), (002), and (541), respectively, the produced MnO_2_ had an α phase. On the other hand, the diffraction peak around 2$\theta$ $\simeq$ 26.0° is associated with the carbon fiber. In Figure 2b, the simulated crystal structure showing the tunnel configuration is presented. Typically, α-MnO_2_ consists of double chains of edge-sharing MnO_6_ octahedra, which are linked at the corners to form 1D 2 × 2 and 1 × 1 tunnels in the tetragonal unit cell. The size of the 2 × 2 tunnel is 4.6 Å, which is a large tunnel for insertion/extraction of cations. Moreover, based on the TEM with selected area electron diffraction (SAED) pattern (Figure 2c,d), the synthesized α-MnO_2_ is a single crystal, as can be inferred from the absence of a ring, which is commonly observed for polycrystalline materials. Distinct spots determined via SAED, which formed hexagonal patterns, are assigned to the family of planes {001}, {00$\overset{\_\_}{1}$}, {541}, {54$\overset{\_\_}{1}$}, {5$\overset{\_\_}{4}\overset{\_\_}{1}$}, and {5$\overset{\_\_}{4}$1}, as can be inferred from the measured angles and distances among these planes. The preferred growth of the crystal is perpendicular to the {001} planes, owing to its high surface energy [53]. Based on the EDS result in Figure S3, the produced α-MnO_2_ nanowires still contain partial amount of K^+^, i.e., 1.32 at %.


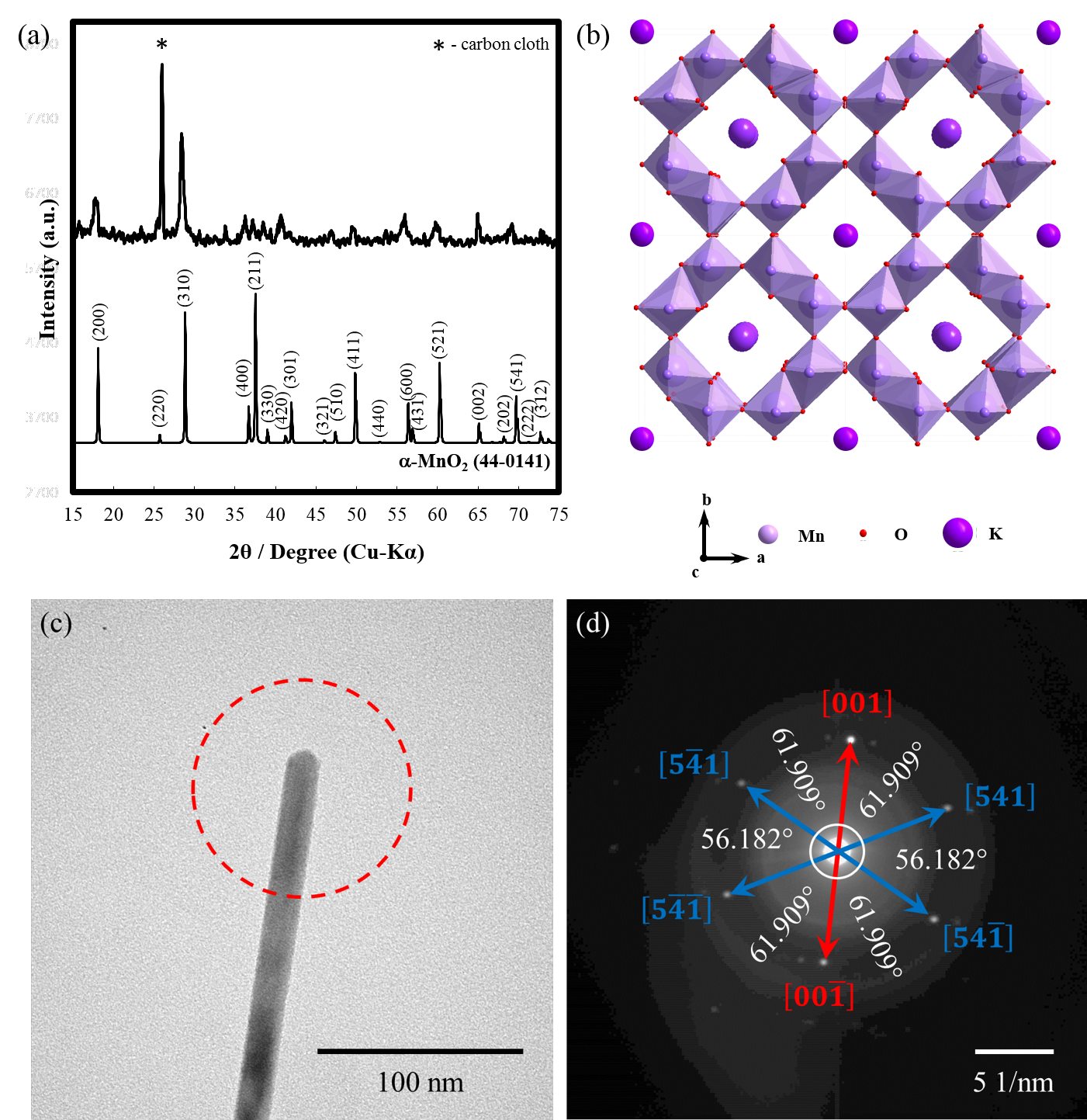


**Figure 2.** (**a**) XRD pattern of synthesized binder-free single crystal α-MnO_2_ nanowire-carbon fiber composite cathode, (**b**) simulated structure, (**c**) TEM image, and (**d**) SAED analysis of single crystal α-MnO_2_.

As shown in Figure 3a, CVs at a scan rate of 0.5 mV/s revealed broad redox peaks around 1.2 and 1.6 V vs. Zn/Zn^2+^ for the sample 0.1 M aqueous Zn(OTf)_2_ electrolyte. The ill-resolved redox peaks for the sample 0.1 M aqueous Zn(OTf)_2_ electrolyte can be attributed to the capacitive behavior of α-MnO_2_ on neutral aqueous electrolyte, i.e., at low salt concentration [54]. Interestingly, when concentration increased to 3.0 M, using the same aqueous electrolyte, well-defined redox peaks were observed at 1.35 and 1.65 V, which were at higher potentials in comparison to those observed at lower concentration. Typically, redox peaks are attributed to Zn^2+^ intercalation/deintercalation, which vary depending on the charge state of Mn [46,52,53]. On the other hand, using 0.1 M Zn(OTf)_2_ electrolyte in DMSO, the CV displayed distinct reversible redox peaks situated around 1.15 and 1.7 V. Since this report is the first which deals with α-MnO_2_ in DMSO-based ZIBs, the charge storage mechanism of the cathode at different scan rates of 0.5, 2.0, 5.0, and 10.0 mV/s were examined, as shown in Figure 3b–d, according to Equations (1) and (2) [55]:

| $i=av^{b}$ | (1) |
| --- | --- |
| $i=k_{1}v+k_{2}v^{1/2}$ | (2) |
| $i/v^{1/2}=k_{1}v^{1/2}+k_{2}$ | (3) |

The *i* and *v* in Equation (1) correspond to the peak current and scan rate, respectively. The *b*-value can be calculated using the slope of log (*v*) vs. log (*i*): if the *b*-value is close to 0.5, the electrochemical behavior is controlled by the diffusion process, while the *b*-value close to 1.0 is based on capacitive behavior. As shown in Figure 3c, the calculated *b*-value is 0.69. It can be inferred that at the considered scan rates, the charge storage mechanism is dominantly diffusion-controlled in 0.1 M Zn(TOf)_2_ DMSO. To understand the behavior with respect to each applied current density, Equation (2) was used, where the $k_{1}v$ term refers to the capacitive process and the $k_{2}v^{1/2}$ term corresponds to the diffusion-controlled process. The values of $k_{1}$ and $k_{2}$ were obtained by using the slope and y-intercept of Equation (3). It is noted that the capacitive contribution of the storage mechanism in DMSO-based ZIBs increased at an incremental scan rate, as shown in Figure 3d. Specifically, the diffusion-controlled process turned out to be the predominant role under lower current densities whilst the capacitive process dominated at higher current densities.


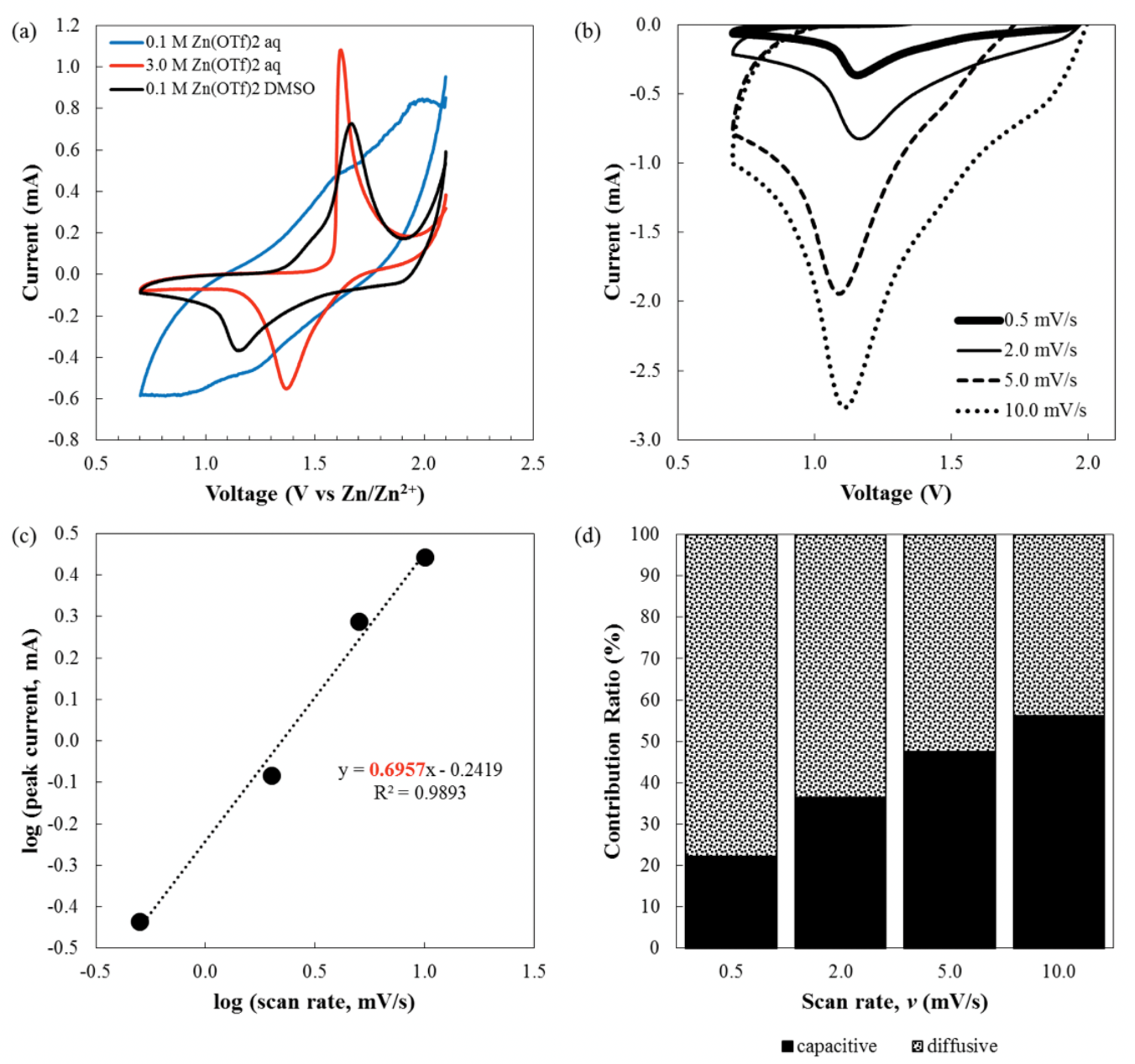


**Figure 3.** (**a**) Cyclic voltammograms of α-MnO_2_ in 0.1 M aqueous Zn(OTf)_2_ solution (blue), 3.0 M aqueous Zn(OTf)_2_ solution (black) and 0.1 M nonaqueous Zn(OTf)_2_ solution (red) at a scan rate of 0.5 mV/s, (**b**) cathodic peaks at different scan rates for α-MnO_2_ with 0.1 M Zn(OTf)_2_ in DMSO, (**c**) linear plot for determination of *b* value for α-MnO_2_ with 0.1 M Zn(OTf)_2_ in DMSO, and (**d**) histogram of capacitive and diffusive contribution for α-MnO_2_ with 0.1 M Zn(OTf)_2_ in DMSO at different scan rates.

The galvanostatic charge-discharge test revealed that the battery using 0.1 M aqueous Zn(OTf)_2_ electrolyte demonstrated higher capacity in comparison to both the 3.0 M aqueous Zn(OTf)_2_ and 0.1 M Zn(OTf)_2_ DMSO for all current densities (50, 100, 150, and 200 mA g^−1^). However, after 56 cycles, the battery eventually failed. In an aqueous system, cycling performance is commonly attributed to the following: 1) dendritic Zn deposition [56], 2) irreversible surface passivation on the Zn anode [57], and 3) dissolution and irreversible phase transformation of the MnO_2_ cathode leading to capacity fading [58]. It is observed that when electrolyte concentration increased to 3.0 M Zn(OTf)_2_, long-term cyclability significantly improved and even reached up to 1000 cycles, although both capacity and capacity retention were found to be relatively lower in comparison to the more dilute aqueous electrolyte system. Based on this result, it is noted that when a concentrated electrolyte was used, anode passivation and dendritic Zn formation could be prevented, but capacity fading remained a critical issue. Hence, replacing the aqueous electrolyte system with a DMSO electrolyte proved to be beneficial in solving the problems of irreversible reactions in the anode and cathode.

In Figure 4, both the galvanostatic charge-discharge test and the cycling performance of the nonaqueous α-MnO_2_-based ZIB with 0.1 M Zn(OTf)_2_ DMSO electrolyte are indicated. For the first 300 cycles, results demonstrated stable capacity of around 60 mAh g^-1^. Thereafter, it stabilized at a capacity of around 50 mAh g^−1^ up to 2000 cycles, at current density of 100 mA g^−1^. It is recognized that the electrochemical performance and cyclability of a ZIB is highly dependent on the stability and reversibility of the reaction, occurring in both anode and cathode. In an aqueous system, efforts are made to avoid the formation of a passivation layer and other irreversible reactions. This is achieved with the use of a mild acidic and neutral pH electrolyte system. However, long-term stability and cyclability are still an imminent issue for an aqueous system due to corrosion and hydrogen evolution problems, which are unavoidable owing to the presence of water. An attempt to address this issue was demonstrated in previous reports [46,52], such as the use of DES utilizing δ-MnO_2_ as cathode material in both unannealed and annealed conditions. The obtained capacity was much lower than that obtained in an aqueous system because of inferior ionic conductivity. However, a stable capacity up to 150 cycles was achieved, which served as the benchmark for this newly explored nonaqueous system. Unfortunately, long-term stability and cyclability beyond 150 cycles was not attained using the DES electrolyte system. This was probably due to an inferior choice of cathode material: δ-MnO_2_, which has a physiosorbed interlayer and structurally bonded water that can affect the reversibility of the reaction in the cathode. Hence, to avoid this problem, α-MnO_2_, having no bonded water, was synthesized and investigated in a novel nonaqueous electrolyte system using DMSO as an electrolyte. In the present case, it can be argued that the long-term cyclability and stability of MnO_2_-based ZIB is not only highly dependent on the cathode and the anode, but also on the electrolyte used. For instance, when α-MnO_2_ was used in an aqueous system, the battery instantly failed after only a few cycles. However, this did not occur when DMSO was used as the electrolyte, even at dilute Zn(OTf)_2_. As shown in Figure S4, both Nyquist and Bode plots of EIS depicted lower impedance value in the anode in the case of the DMSO-based ZIBs compared to the aqueous ones. This implied that DMSO played a significant role in the prevention of Zn passivation, dendritic Zn deposition, and irreversible phase transformation of MnO_2_, leading to better cyclability and electrochemical performance as shown in Figure S5.


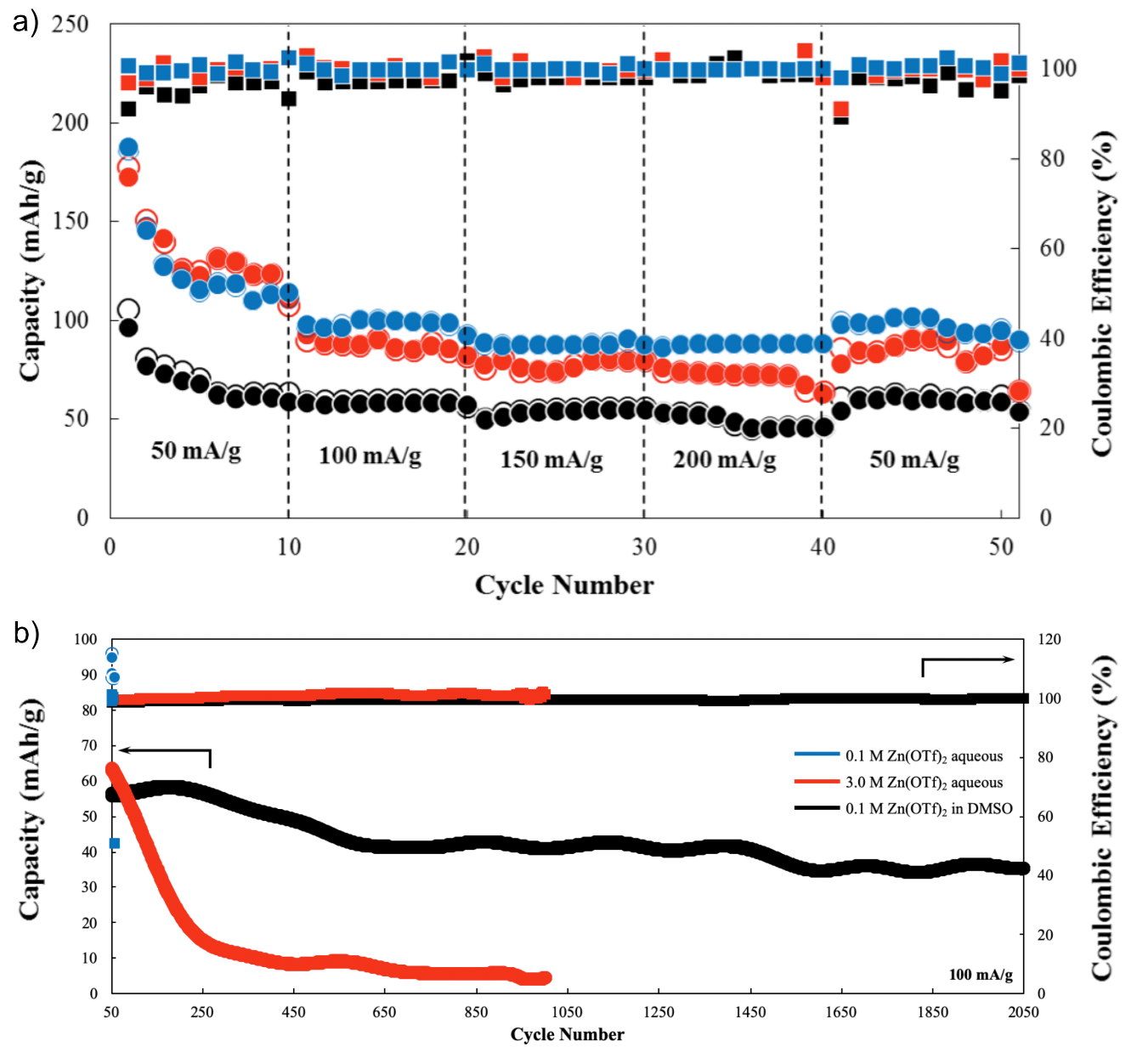


**Figure 4.** (**a**) Rate capability test of α-MnO_2_-carbon fiber composite at different current densities of 50, 100, 150, and 200 mA·g^−1^ and the corresponding voltage-charge profile with voltage window of 0.4 to 1.9 V, and (**b**) galvanostatic charge-discharge test of α-MnO_2_-carbon fiber composite at current density of 100 mA·g^−1^ in aqueous and DMSO based electrolyte.

4. Conclusions

It is evident that binder-free α-MnO_2_ nanowires on carbon cloth was successfully prepared via the hydrothermal method. In both aqueous and DMSO-based electrolytes, the characteristics and electrochemical performances of the cathode were examined. It was found that the ZIB using 0.1 M Zn(OTf)_2_ DMSO electrolyte demonstrated exceptional cycling performance in comparison to the 0.1 M and 3.0 M Zn(OTf)_2_ aqueous electrolytes. In the DMSO electrolyte, even in the absence of a polymeric binder, the cathode proved to be very stable. Overall, higher cyclability and stability of the MnO_2_-based ZIBs could be attained with the Zn(OTf)_2_ DMSO electrolyte.

**Supplementary Materials:** Supplementary materials can be found at www.mdpi.com/1422-0067/21/9/3113/s1. Figure S1. Morphology of MnO_2_-carbon fiber composite synthesized at 140 °C with respect to synthesis time: (a) 24 h. (b) 72 h. and (c) 168 h. Figure S2. Morphology of MnO_2_-carbon fiber composite with respect to synthesis temperature: (a) 110 °C (b) 140 °C and (c) 180 °C. Figure S3. EDS analysis of synthesized α-MnO2 nanowire-carbon fiber composite. Figure S4. (a) Nyquist and (b) Bode plots of EIS of the zinc-ion batteries using 0.1 M and 3.0 M aqueous Zn(OTf)_2_ and 0.1 M Zn(OTf)_2_ DMSO electrolytes. Figure S5. SEM image of Zn anode in DMSO-based ZIB after more than 2000 cycles.

**Author Contributions:** Conceptualization, S.K.; methodology, R.D.C. and S.K.; investigation: R.D.C. and L.M.D.; formal analysis, R.D.C. and S.K.; writing-original draft preparation, R.D.C. and S.K.; writing—review and editing, R.D.C., L.M.D., M.T.N., T.Y., H.W., A.S. and S.K.; supervision, S.K.; funding acquisition, S.K.; project administration, S.K. All authors have read and agreed to the published version of the manuscript.

**Funding:** The Energy Conservation Promotion Fund from the Energy Policy and Planning Office, Ministry of Energy, Grant for Join Funding, Ratchadaphiseksomphot Endowment Fund, and the Energy Storage Cluster of Chulalongkorn University are acknowledged.

**Acknowledgments:** The authors thank Sigurd Skogestad for his comments and the support from Ratchadaphiseksomphot Endowment Fund, Chulalongkorn University.

**Conflicts of Interest:** The authors declare no conflict of interest.

Abbreviations

| ZIB | Zinc-ion battery |
| --- | --- |
| DMSO | Dimethyl sulfoxide |
| LIB | Lithium-ion battery |
| rGO | Reduced graphene oxide |
| CNT | Carbon nanotube |
| NMP | N-methyl-2-pyrrolidone |
| DES | Deep eutectic solvent |
| DI water | Deionized water |
| CV | Cyclic voltammetry |
| EIS | Electrochemical Impedance Spectroscopy |
| OCV | Open circuit voltage |
| SEM | Scanning electron microscope |
| TEM | Transmission electron microscope |
| XRD | X-ray diffraction |
| EDS | Energy dispersive X-Ray spectroscopy |
| SAED | Selected area electron diffraction |

References

1. Wu, X.; Song, K.; Zhang, X.; Hu, N.; Li, L.; Li, W.; Zhang, L.; Zhang, H. Safety Issues in Lithium Ion Batteries: Materials and Cell Design. *Front. Energy Res.* **2019**, *7*, doi:10.3389/fenrg.2019.00065.
2. Ouyang, D.; Chen, M.; Huang, Q.; Weng, J.; Wang, Z.; Wang, J. A Review on the Thermal Hazards of the Lithium-Ion Battery and the Corresponding Countermeasures. *Appl. Sci.* **2019**, *9*, 2483, doi:10.3390/app9122483.
3. Ould Ely, T.; Kamzabek, D.; Chakraborty, D. Batteries Safety: Recent Progress and Current Challenges. *Front. Energy Res.* **2019**, *7*, doi:10.3389/fenrg.2019.00071.
4. Su, B.; Zhang, J.; Fujita, M.; Zhou, W.; Sit, P.H.-L.; Yu, D.Y.W. Na2SeO3: A Na-Ion Battery Positive Electrode Material with High Capacity. *J. Electrochem. Soc.* **2019**, *166*, A5075–A5080, doi:10.1149/2.0131903jes.
5. Zhang, R.; Tutusaus, O.; Mohtadi, R.; Ling, C. Magnesium-Sodium Hybrid Battery With High Voltage, Capacity and Cyclability. *Front. Chem.* **2018**, *6*, doi:10.3389/fchem.2018.00611.
6. Leisegang, T.; Meutzner, F.; Zschornak, M.; Münchgesang, W.; Schmid, R.; Nestler, T.; Eremin, R.A.; Kabanov, A.A.; Blatov, V.A.; Meyer, D.C. The Aluminum-Ion Battery: A Sustainable and Seminal Concept? *Front. Chem.* **2019**, *7*, doi:10.3389/fchem.2019.00268.
7. Mokhtar, M.; Talib, M.Z.M.; Majlan, E.H.; Tasirin, S.M.; Ramli, W.M.F.W.; Daud, W.R.W.; Sahari, J. Recent developments in materials for aluminum–air batteries: A review. *J. Ind. Eng. Chem.* **2015**, *32*, 1–20, doi:10.1016/j.jiec.2015.08.004.
8. Ling, C.; Zhang, R. Manganese Dioxide As Rechargeable Magnesium Battery Cathode. *Front. Energy Res.* **2017**, *5*, doi:10.3389/fenrg.2017.00030.
9. Shrestha, N.; Raja, K.S.; Utgikar, V. Mg-RE Alloy Anode Materials for Mg-Air Battery Application. *J. Electrochem. Soc.* **2019**, *166*, A3139–A3153, doi:10.1149/2.0131914jes.
10. Tian, H.; Gao, T.; Li, X.; Wang, X.; Luo, C.; Fan, X.; Yang, C.; Suo, L.; Ma, Z.; Han, W.; et al. High power rechargeable magnesium/iodine battery chemistry. *Nat. Commun.* **2017**, *8*, 14083, doi:10.1038/ncomms14083.
11. Lao-atiman, W.; Julaphatachote, T.; Boonmongkolras, P.; Kheawhom, S. Printed Transparent Thin Film Zn-MnO2 Battery. *J. Electrochem. Soc.* **2017**, *164*, A859–A863, doi:10.1149/2.1511704jes.
12. De Juan-Corpuz, L.M.; Corpuz, R.D.; Somwangthanaroj, A.; Nguyen, M.T.; Yonezawa, T.; Ma, J.; Kheawhom, S. Binder-Free Centimeter-Long V2O5 Nanofibers on Carbon Cloth as Cathode Material for Zinc-Ion Batteries. *Energies* **2019**, *13*, 31, doi:10.3390/en13010031.
13. Hosseini, S.; Abbasi, A.; Uginet, L.-O.; Haustraete, N.; Praserthdam, S.; Yonezawa, T.; Kheawhom, S. The Influence of Dimethyl Sulfoxide as Electrolyte Additive on Anodic Dissolution of Alkaline Zinc-Air Flow Battery. *Sci. Rep.* **2019**, *9*, 14958, doi:10.1038/s41598-019-51412-5.
14. Yang, D.; Tan, H.; Rui, X.; Yu, Y. Electrode Materials for Rechargeable Zinc-Ion and Zinc-Air Batteries: Current Status and Future Perspectives. *Electrochem. Energy Rev.* **2019**, *2*, 395–427, doi:10.1007/s41918-019-00035-5.
15. Konarov, A.; Voronina, N.; Jo, J.H.; Bakenov, Z.; Sun, Y.-K.; Myung, S.-T. Present and Future Perspective on Electrode Materials for Rechargeable Zinc-Ion Batteries. *ACS Energy Lett.* **2018**, *3*, 2620–2640, doi:10.1021/acsenergylett.8b01552.
16. Abbasi, A.; Hosseini, S.; Somwangthanaroj, A.; Mohamad, A.A.; Kheawhom, S. Poly(2,6-Dimethyl-1,4-Phenylene Oxide)-Based Hydroxide Exchange Separator Membranes for Zinc–Air Battery. *Int. J. Mol. Sci.* **2019**, *20*, 3678, doi:10.3390/ijms20153678.
17. Yao, S.; Sun, X.; Xiao, M.; Cheng, J.; Shen, Y. Equivalent Circuit Model Construction and Dynamic Flow Optimization Based on Zinc–Nickel Single-Flow Battery. *Energies* **2019**, *12*, 582, doi:10.3390/en12040582.
18. Hosseini, S.; Han, S.J.; Arponwichanop, A.; Yonezawa, T.; Kheawhom, S. Ethanol as an electrolyte additive for alkaline zinc-air flow batteries. *Sci. Rep.* **2018**, *8*, 11273, doi:10.1038/s41598-018-29630-0.
19. Poolnapol, L.; Kao-ian, W.; Somwangthanaroj, A.; Mahlendorf, F.; Nguyen, M.T.; Yonezawa, T.; Kheawhom, S. Silver Decorated Reduced Graphene Oxide as Electrocatalyst for Zinc–Air Batteries. *Energies* **2020**, *13*, 462, doi:10.3390/en13020462.
20. Xie, C.; Liu, Y.; Lu, W.; Zhang, H.; Li, X. Highly stable zinc–iodine single flow batteries with super high energy density for stationary energy storage. *Energy Environ. Sci.* **2019**, *12*, 1834–1839, doi:10.1039/C8EE02825G.
21. Selverston, S.; Savinell, R.F.; Wainright, J.S. Zinc-Iron Flow Batteries with Common Electrolyte. *J. Electrochem. Soc.* **2017**, *164*, A1069–A1075, doi:10.1149/2.0591706jes.
22. Liu, J.; Xu, C.; Chen, Z.; Ni, S.; Shen, Z.X. Progress in aqueous rechargeable batteries. *Green Energy Environ.* **2018**, *3*, 20–41, doi:10.1016/j.gee.2017.10.001.
23. Ao, H.; Zhao, Y.; Zhou, J.; Cai, W.; Zhang, X.; Zhu, Y.; Qian, Y. Rechargeable aqueous hybrid ion batteries: Developments and prospects. *J. Mater. Chem. A* **2019**, *7*, 18708–18734, doi:10.1039/C9TA06433H.
24. Van Genderen, E.; Wildnauer, M.; Santero, N.; Sidi, N. A global life cycle assessment for primary zinc production. *Int. J. Life Cycle Assess.* **2016**, *21*, 1580–1593, doi:10.1007/s11367-016-1131-8.
25. Li, H.; Ma, L.; Han, C.; Wang, Z.; Liu, Z.; Tang, Z.; Zhi, C. Advanced rechargeable zinc-based batteries: Recent progress and future perspectives. *Nano Energy* **2019**, *62*, 550–587, doi:10.1016/j.nanoen.2019.05.059.
26. Wang, B.; Han, Y.; Wang, X.; Bahlawane, N.; Pan, H.; Yan, M.; Jiang, Y. Prussian Blue Analogs for Rechargeable Batteries. *iScience* **2018**, *3*, 110–133, doi:10.1016/j.isci.2018.04.008.
27. Zhang, L.; Chen, L.; Zhou, X.; Liu, Z. Towards High-Voltage Aqueous Metal-Ion Batteries Beyond 1.5 V: The Zinc/Zinc Hexacyanoferrate System. *Adv. Energy Mater.* **2015**, *5*, 1400930, doi:10.1002/aenm.201400930.
28. Zhang, L.; Chen, L.; Zhou, X.; Liu, Z. Morphology-Dependent Electrochemical Performance of Zinc Hexacyanoferrate Cathode for Zinc-Ion Battery. *Sci. Rep.* **2015**, *5*, 18263, doi:10.1038/srep18263.
29. Tang, B.; Zhou, J.; Fang, G.; Guo, S.; Guo, X.; Shan, L.; Tang, Y.; Liang, S. Structural Modification of V2O5 as High-Performance Aqueous Zinc-Ion Battery Cathode. *J. Electrochem. Soc.* **2019**, *166*, A480–A486, doi:10.1149/2.0081904jes.
30. Zhou, J.; Shan, L.; Wu, Z.; Guo, X.; Fang, G.; Liang, S. Investigation of V2O5 as a low-cost rechargeable aqueous zinc ion battery cathode. *Chem. Commun.* **2018**, *54*, 4457–4460, doi:10.1039/C8CC02250J.
31. Xie, C.; Li, T.; Deng, C.; Song, Y.; Zhang, H.; Li, X. A highly reversible neutral zinc/manganese battery for stationary energy storage. *Energy Environ. Sci.* **2020**, *13*, 135–143, doi:10.1039/C9EE03702K.
32. Julien, M.C.; Mauger, A. Nanostructured MnO2 as Electrode Materials for Energy Storage. *Nanomaterials* **2017**, *7*, 396, doi:10.3390/nano7110396.
33. Hosseini, S.; Lao-atiman, W.; Han, S.J.; Arpornwichanop, A.; Yonezawa, T.; Kheawhom, S. Discharge Performance of Zinc-Air Flow Batteries Under the Effects of Sodium Dodecyl Sulfate and Pluronic F-127. *Sci. Rep.* **2018**, *8*, 14909, doi:10.1038/s41598-018-32806-3.
34. Valipour, A.; Hamnabard, N.; Meshkati, S.M.H.; Pakan, M.; Ahn, Y.-H. Effectiveness of phase- and morphology-controlled MnO2 nanomaterials derived from flower-like δ-MnO2 as alternative cathode catalyst in microbial fuel cells. *Dalton Trans.* **2019**, *48*, 5429–5443, doi:10.1039/C9DT00520J.
35. Lao-atiman, W.; Olaru, S.; Arpornwichanop, A.; Kheawhom, S. Discharge performance and dynamic behavior of refuellable zinc-air battery. *Sci. Data* **2019**, *6*, 168, doi:10.1038/s41597-019-0178-3.
36. Wang, L.; Wu, Q.; Abraham, A.; West, P.J.; Housel, L.M.; Singh, G.; Sadique, N.; Quilty, C.D.; Wu, D.; Takeuchi, E.S.; et al. Silver-Containing α-MnO2 Nanorods: Electrochemistry in Rechargeable Aqueous Zn-MnO2 Batteries. *J. Electrochem. Soc.* **2019**, *166*, A3575–A3584, doi:10.1149/2.0101915jes.
37. Singh, S.; Sahoo, R.K.; Shinde, N.M.; Yun, J.M.; Mane, R.S.; Kim, K.H. Synthesis of Bi2O3-MnO2 Nanocomposite Electrode for Wide-Potential Window High Performance Supercapacitor. *Energies* **2019**, *12*, 3320, doi:10.3390/en12173320.
38. Wu, D.; Xie, X.; Zhang, Y.; Zhang, D.; Du, W.; Zhang, X.; Wang, B. MnO2/Carbon Composites for Supercapacitor: Synthesis and Electrochemical Performance. *Front. Mater.* **2020**, *7*, doi:10.3389/fmats.2020.00002.
39. Wang, J.; Wang, J.-G.; Liu, H.; Wei, C.; Kang, F. Zinc ion stabilized MnO2 nanospheres for high capacity and long lifespan aqueous zinc-ion batteries. *J. Mater. Chem. A* **2019**, *7*, 13727–13735, doi:10.1039/C9TA03541A.
40. Pan, H.; Shao, Y.; Yan, P.; Cheng, Y.; Han, K.S.; Nie, Z.; Wang, C.; Yang, J.; Li, X.; Bhattacharya, P.; et al. Reversible aqueous zinc/manganese oxide energy storage from conversion reactions. *Nat. Energy* **2016**, *1*, 16039, doi:10.1038/nenergy.2016.39.
41. Khamsanga, S.; Pornprasertsuk, R.; Yonezawa, T.; Mohamad, A.A.; Kheawhom, S. δ-MnO2 nanoflower/graphite cathode for rechargeable aqueous zinc ion batteries. *Sci. Rep.* **2019**, *9*, 8441, doi:10.1038/s41598-019-44915-8.
42. Huang, Y.; Liu, J.; Huang, Q.; Zheng, Z.; Hiralal, P.; Zheng, F.; Ozgit, D.; Su, S.; Chen, S.; Tan, P.-H.; et al. Flexible high energy density zinc-ion batteries enabled by binder-free MnO2/reduced graphene oxide electrode. *NPJ Flex. Electron.* **2018**, *2*, 21, doi:10.1038/s41528-018-0034-0.
43. Xu, D.; Li, B.; Wei, C.; He, Y.-B.; Du, H.; Chu, X.; Qin, X.; Yang, Q.-H.; Kang, F. Preparation and Characterization of MnO2/acid-treated CNT Nanocomposites for Energy Storage with Zinc Ions. *Electrochim. Acta* **2014**, *133*, 254–261, doi:10.1016/j.electacta.2014.04.001.
44. Jiao, T.; Yang, Q.; Wu, S.; Wang, Z.; Chen, D.; Shen, D.; Liu, B.; Cheng, J.; Li, H.; Ma, L.; et al. Binder-free hierarchical VS2 electrodes for high-performance aqueous Zn ion batteries towards commercial level mass loading. *J. Mater. Chem. A* **2019**, *7*, 16330–16338, doi:10.1039/C9TA04798K.
45. Yin, B.; Zhang, S.; Ke, K.; Xiong, T.; Wang, Y.; Lim, B.K.D.; Lee, W.S.V.; Wang, Z.; Xue, J. Binder-free V2O5/CNT paper electrode for high rate performance zinc ion battery. *Nanoscale* **2019**, *11*, 19723–19728, doi:10.1039/C9NR07458A.
46. Corpuz, R.D.; De Juan, L.M.Z.; Praserthdam, S.; Pornprasertsuk, R.; Yonezawa, T.; Nguyen, M.T.; Kheawhom, S. Annealing induced a well-ordered single crystal δ-MnO2 and its electrochemical performance in zinc-ion battery. *Sci. Rep.* **2019**, *9*, 15107, doi:10.1038/s41598-019-51692-x.
47. Demir-Cakan, R.; Palacin, M.R.; Croguennec, L. Rechargeable aqueous electrolyte batteries: From univalent to multivalent cation chemistry. *J. Mater. Chem. A* **2019**, *7*, 20519–20539, doi:10.1039/C9TA04735B.
48. Wu, Y.; Zhang, K.; Chen, S.; Liu, Y.; Tao, Y.; Zhang, X.; Ding, Y.; Dai, S. Proton Inserted Manganese Dioxides as a Reversible Cathode for Aqueous Zn-Ion Batteries. *ACS Appl. Energy Mater.* **2020**, *3*, 319–327, doi:10.1021/acsaem.9b01554.
49. Zhang, N.; Dong, Y.; Wang, Y.; Wang, Y.; Li, J.; Xu, J.; Liu, Y.; Jiao, L.; Cheng, F. Ultrafast Rechargeable Zinc Battery Based on High-Voltage Graphite Cathode and Stable Nonaqueous Electrolyte. *ACS Appl. Mater. Interfaces* **2019**, *11*, 32978–32986, doi:10.1021/acsami.9b10399.
50. Xu, K. Nonaqueous Liquid Electrolytes for Lithium-Based Rechargeable Batteries. *Chem. Rev.* **2004**, *104*, 4303–4418, doi:10.1021/cr030203g.
51. Zhu, G.; Angell, M.; Pan, C.-J.; Lin, M.-C.; Chen, H.; Huang, C.-J.; Lin, J.; Achazi, A.J.; Kaghazchi, P.; Hwang, B.-J.; et al. Rechargeable aluminum batteries: Effects of cations in ionic liquid electrolytes. *RSC Adv.* **2019**, *9*, 11322–11330, doi:10.1039/C9RA00765B.
52. Kao-ian, W.; Pornprasertsuk, R.; Thamyongkit, P.; Maiyalagan, T.; Kheawhom, S. Rechargeable Zinc-Ion Battery Based on Choline Chloride-Urea Deep Eutectic Solvent. *J. Electrochem. Soc.* **2019**, *166*, A1063–A1069, doi:10.1149/2.0641906jes.
53. Chen, B.-R.; Sun, W.; Kitchaev, D.A.; Mangum, J.S.; Thampy, V.; Garten, L.M.; Ginley, D.S.; Gorman, B.P.; Stone, K.H.; Ceder, G.; et al. Understanding crystallization pathways leading to manganese oxide polymorph formation. *Nat. Commun.* **2018**, *9*, 2553, doi:10.1038/s41467-018-04917-y.
54. Qu, Q.; Zhang, P.; Wang, B.; Chen, Y.; Tian, S.; Wu, Y.; Holze, R. Electrochemical Performance of MnO_2_ Nanorods in Neutral Aqueous Electrolytes as a Cathode for Asymmetric Supercapacitors. *J. Phys. Chem. C* **2009**, *113*, 14020–14027, doi:10.1021/jp8113094.
55. Wang, J.; Polleux, J.; Lim, J.; Dunn, B. Pseudocapacitive Contributions to Electrochemical Energy Storage in TiO2 (Anatase) Nanoparticles. *J. Phys. Chem. C* **2007**, *111*, 14925–14931, doi:10.1021/jp074464w.
56. Zhao, Z.; Zhao, J.; Hu, Z.; Li, J.; Li, J.; Zhang, Y.; Wang, C.; Cui, G. Long-life and deeply rechargeable aqueous Zn anodes enabled by a multifunctional brightener-inspired interphase. *Energy Environ. Sci.* **2019**, *12*, 1938–1949, doi:10.1039/C9EE00596J.
57. Xu, W.; Wang, Y. Recent Progress on Zinc-Ion Rechargeable Batteries. *Nano-Micro Lett.* **2019**, *11*, 90, doi:10.1007/s40820-019-0322-9.
58. Zhang, N.; Cheng, F.; Liu, J.; Wang, L.; Long, X.; Liu, X.; Li, F.; Chen, J. Rechargeable aqueous zinc-manganese dioxide batteries with high energy and power densities. *Nat. Commun.* **2017**, *8*, 405, doi:10.1038/s41467-017-00467-x.

| 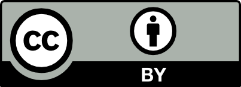 | © 2020 by the authors. Licensee MDPI, Basel, Switzerland. This article is an open access article distributed under the terms and conditions of the Creative Commons Attribution (CC BY) license (http://creativecommons.org/licenses/by/4.0/). |
| --- | --- |
